# How effective is tetracaine 4% gel, before a venipuncture, in reducing procedural pain in infants: a randomized double-blind placebo controlled trial

**DOI:** 10.1186/1471-2431-7-7

**Published:** 2007-02-08

**Authors:** Brigitte Lemyre, Debora L Hogan, Isabelle Gaboury, Rebecca Sherlock, Colline Blanchard, David Moher

**Affiliations:** 1 Department of Pediatrics, University of Ottawa, Ottawa, Canada; 2 Department of Nursing, Children's Hospital of Eastern Ontario, Ottawa, Canada; 3 Chalmers Research Group, Children's Hospital of Eastern Ontario Research Institute, Ottawa, Canada; 4 Department of Pediatrics, BC Children's and Women's Health Center, Vancouver, Canada; 5 Department of Healthcare and Epidemiology, Faculty of Medicine, University of British Columbia, Vancouver, Canada; 6 Department of Pharmacy, Children's Hospital of Eastern Ontario, Ottawa, Canada; 7 Department of Epidemiology & Community Medicine, Faculty of Medicine, University of Ottawa, Ottawa, Canada

## Abstract

**Background:**

Procedural pain relief is sub-optimal in neonates. Topical tetracaine provides pain relief in children. Evidence of its efficacy and safety in neonates is limited. The objective of this study was to assess the efficacy and safety of topical tetracaine on the pain response of neonates during a venipuncture.

**Methods:**

Medically stable infants greater than or equal to 24 weeks gestation, requiring a venipuncture, were included. Following randomization and double blinding, 1.1 g of tetracaine or placebo was applied to the skin for 30 minutes. Participants received oral sucrose if they met local eligibility criteria. The venipuncture was performed according to a standard protocol. A medium effect size in the pain score (corresponding to about 2 point difference in the PIPP score) was considered clinically significant, leading to a sample size of 142 infants, with 80% statistical power. Local skin reactions and immediate adverse cardiorespiratory events were noted. The primary outcome, PIPP score at 1 minute, was analysed using an independent Student's t-test.

**Results:**

One hundred and forty two infants were included, 33 +/- 4 weeks gestation, 2100 +/- 900 grams and 6 +/- 3 days of age. There was almost no difference in PIPP scores at 1 minute between groups (mean difference -0.09; 95% confidence interval [CI]: -1.68 to 1.50; P = . 91). Similarly, there were no differences in PIPP scores during the 2^nd^, 3^rd ^and 4th minute. Duration of cry did not differ between the groups (median difference, 0; 95% CI, -3 to 0; P = . 84). The majority of infants in both groups received sucrose 24%. Sucrose had a significant effect on the PIPP score, as assessed by an ANOVA model (p = 0.0026). Local skin erythema was observed transiently in 11 infants (7 in the tetracaine and 4 in the placebo group). No serious side effect was observed.

**Conclusion:**

Tetracaine did not significantly decrease procedural pain in infants undergoing a venipuncture, when used in combination with routine sucrose administration.

## Background

Neonates admitted to neonatal intensive care units (NICUs) undergo multiple painful procedures during their hospitalizations. The most prevalent of these procedures is blood sampling either by heel lancing or venipuncture. Infants born prematurely experience an average of 134 painful procedures within the first two weeks of life or up to 14 painful procedures a day [1,2].

It is well established that neonates experience pain [3,4]. Pain is stressful and leads to behavioural and physiologic disorganization, which in turn may lead to several long-term consequences, including chronic pain and altered neurobehavioral responses to subsequent pain [3]. Our standard of care at the time this protocol was written was to use non-pharmacological interventions including swaddling and non-nutritive sucking for painful procedures, such as venipuncture and heel pricks. Recently, experts have recommended sucrose and/or local or systemic analgesia before such procedures [5]. Side effects of systemic analgesics limit their use on a regular basis [6,7].

EMLA (Lidocaine-Prilocaine 5% cream), a widely used anesthetic cream, has a relatively long onset of action (60 minutes), can cause local blanching and vasoconstriction and is associated with a theoretical risk of methemoglobinemia, which has been reported in infants in a few case reports [8,9]. This can limit its usefulness in newborns.

Tetracaine gel (4% w/w tetracaine in an aqueous gel, Amethocaine gel or Ametop, Smith-Nephew Inc, St-Laurent, Quebec), a topical anesthetic developed in the early 1990s acts by blocking the flux of sodium ions across the axon and, thus, blocking the action potential. Due to its intrinsic properties, it appears to be a good option for newborns. First, its onset of action is only 30 to 45 minutes. Second, it has no risk of methemoglobinemia. In addition, it causes local vasodilatation, which may be an advantage in newborns as it can be a challenge to obtain IV access or blood. Several trials involving older children have demonstrated the efficacy of tetracaine in decreasing pain associated with venipuncture, subcutaneous vaccination and Port-a-Cath puncture [10-16].

The localized anesthetic action of tetracaine has been demonstrated in infants after 30 minutes [17]. Two randomized controlled trials (RCTs) found a decrease in pain scores and in duration of cry in infants who received tetracaine before a venipuncture or a venous catheter insertion, however only 80 infants in total were enrolled and very low birth weight (VLBW) infants were not included [18,19]. No benefit was found for heel pricks or peripherally inserted central catheter in a similar population in four other randomized trials [7,20-23].

The evidence regarding safety and efficacy of tetracaine in infants is limited by small numbers of infants enrolled in trials and exclusion of VLBW infants. The objective of this study was to evaluate whether the application of tetracaine 4% before a venipuncture in newborn infants would safely and significantly decrease procedural pain as compared to placebo.

## Methods

### Participants

Infants admitted to the Neonatal Intensive Care Unit (NICU) of The Ottawa Hospital, General Campus (25 bed level 2–3 unit) or The Children's Hospital of Eastern Ontario (20 bed level 3a unit), Ottawa, Canada between January 2003 and December 2004 were eligible for this study if they required venous blood work. Infants were enrolled if they met the following inclusion criteria: (1) born at ≥ 24 weeks gestation, (2) skin considered in good condition (no burns or rash), (3) if < 27 weeks gestation, at least 48 h of life and (4) considered stable by the attending neonatologist.

### Eligibility criteria

Infants were excluded if they met any of the following criteria: (1) skin considered immature (insensible water losses requiring more fluids than usual for gestation), (2) suspected or proven significant central nervous system anomaly, (3) receiving opioids or sedatives at time of venipuncture or in the previous 12 hours or receiving muscle relaxants, (4) facial anomalies (cleft lip, Moebius syndrome) preventing typical facial expression of pain or (5) sub-optimal hepatic function (ALT > 2× upper normal limit) or sub-optimal renal function (urine output < 1 ml/kg/hour in the 12 hours prior to the research intervention).

The study protocol was approved by the Research Ethics Boards of The Ottawa Hospital and the Children's Hospital of Eastern Ontario. Written informed consent was obtained from a parent or legal guardian for all enrolled infants.

### Intervention

Under the supervision of the research assistant, the study gel was applied to the proposed venipuncture site 30 minutes prior to the venipuncture, by a nurse 'blind' to group assignment. Tetracaine 4% gel was applied to the skin of each participant allocated to the treatment group; Professional Care Lotion (by Smith-Nephew), a skin moisturizer was applied to the skin of each participant allocated to the placebo group. An occlusive dressing (sterile Saran Wrap) was applied over the gel. Tubes of 1.5 g of tetracaine were used, of which 1.1 g could be extracted; the dose of gel applied to the skin was therefore 1.1 g. After 30 minutes, the gel was removed and the venipuncture began.

### Blinding

The study gels were packaged by a single research pharmacist in identical looking ointment jars identified by medication numbers, which matched the enrolment numbers. Both gels were white, odorless and had the same consistency. Pre-prepared jars containing tetracaine 4% or placebo gel identified by the medication numbers were sent to the NICUs and stored in a climate-controlled refrigerator. Once a participant was enrolled, the next sequential jar was used for the venipuncture. The parents, guardians and research team, including outcome assessors, were blind to treatment assignment throughout the study. The success of our blinding procedure was not assessed.

### Randomization

Infants were randomized having equal probability (1:1) of being allocated to either placebo or tetracaine 4% gel. Since the infants' gestational age and maturity varied across the two NICUs, the randomization was stratified by centre. The sequence, a random-permuted block with block size of 4, was computer generated by the study statistician. To help ensure adequate allocation concealment, the sequence was kept centrally on computer and could only be activated when there was an eligible baby to randomize. Only the research pharmacist was aware of the randomization code. The randomization sequence was not broken until data analysis was completed.

### Co-interventions

Where possible, both groups received standard, currently practiced non-pharmacological measures of non-nutritive sucking, swaddling and comforting throughout the procedure, as appropriate for gestation. Between the time the study protocol was written and funded and the beginning of the trial, it became standard policy in both NICUs that infants undergoing painful procedures receive oral sucrose 24% before such procedures, depending on gestational age and ability to feed. Withholding the standard of care for study patients is unethical, thus infants enrolled in the trial received sucrose if they met sucrose eligibility criteria: 26^0 ^to 32^6 ^weeks receiving minimum 50% of oral/enteral feeds, > 32 weeks receiving any amount of oral/enteral feeds, infants on full feeds regardless of gestational age, suck and/or swallow reflex present and if intubated, ventilator rate < 20/minute. Sucrose 24% was administered in doses of 0.1 to 0.5 cc depending on gestational age.

### Procedure

The study gel was applied and removed by the nurse (bedside or clinical/team leader) performing the blood work. S/he was unaware of group allocation of the infant. The blood work was performed according to a standardized protocol.

Data was collected during 5 phases of the venipuncture: 1) Application phase – the nurse examined the skin. S/he applied the study gel to the appropriate venipuncture site and an occlusive dressing (sterile Saran wrap) was applied over the site. After 30 minutes the dressing and the gel were removed. 2) Baseline – The infant remained undisturbed in a position of comfort for 1 minute. 3) Preparation – The skin was cleansed with an alcohol or a chlorhexidine swab. 4) Venipuncture – Skin puncture was performed with a #25 gauge Butterfly, using a tourniquet. Bloodwork was performed on the dorsum of the hands or feet or antecubital area. 5) Recovery – The infant was returned to a position of comfort at which point filming ceased.

A maximum of three attempts (at the same site) were allowed before the procedure was stopped and declared unsuccessful.

Physiological indicators of pain (heart rate, blood pressure, respiratory rate, oxygen saturation) were continuously recorded using a Hewlett Packard Neonatal monitor for the phases of recording.

The Research Assistant videotaped facial action indicators from baseline to the end of the recovery phase with a Sony 8 mm digital Camcorder. The total duration of cry from skin puncture to recovery was determined from the video camera recordings. All data was entered on a personal digital assistant and subsequently uploaded on to a central database.

### Outcomes

#### Efficacy

The primary outcome was the Premature Infant Pain Profile (PIPP) score in the first minute after skin puncture. The PIPP is a tool that was specifically developed to assess acute pain in preterm and term infants [24]. It has undergone extensive clinimetric development for procedural pain of heel-stick, venipuncture, intravenous insertion and circumcision. Interrater and intrarater reliability analysis for individual event scores yielded coefficients of 0.93 to 0.96 and 0.94 to 0.98, respectively [24].

One of two trained facial coders from Dr. Bonnie Stevens' laboratory at The Hospital for Sick Children in Toronto assessed the video camera recordings of the venipuncture for three specific validated facial expressions of pain which are part of the PIPP score: brow bulge, nasolabial furrow and eye squeeze. The intrarater and interrater reliability scores of the facial coders were 0.95 and 0.95 (percent agreement) respectively during the study. Coders were blind to the group allocation of the infants. One investigator (DH) calculated PIPP scores every minute, from baseline to the recovery phase, by combining these behavioral findings to the other components of the PIPP. Each PIPP score was derived by calculating two 30-second intervals for each minute of the procedure. The PIPP score was assigned as the highest value of the two 30 second scores.

The secondary outcome measures were: (1) PIPP scores during the second, third and fourth minute of the insertion phase (compared both independently and longitudinally including the observationduring the first minute of the procedure) (2) mean heart rate in beats per minute, mean respiratory rate per minute, mean blood pressure in mm Hg and mean O_2 _saturation in % at the end of baseline, 1 minute after skin puncture then 2, 3, 4, 5 and 10 minutes after skin puncture (3) duration of cry, from skin puncture to recovery (4) mean number of attempts required to obtain the blood work, success rate at obtaining the blood work and subjective measure of easiness on a scale of 1 to 5 (1 being very easy and 5 very hard). Ease of procedure was derived by asking the nurse to appraise his/her assessment of the ease of obtaining bloodwork.

#### Harms

The safety of tetracaine was appraised using the following data: local skin reaction (redness, oedema), complete blood count and differential (pre and post intervention), AST and ALT (pre and post intervention), and creatinine levels (post intervention). Blood work (0.8 cc) was collected along with clinically indicated (by the treating team) blood tests, within 72 hours of the intervention and then again, 48 to 72 hours after the intervention. There is no previously reported blood safety data on tetracaine in neonates and so we monitored the function of important organs (bone marrow, liver, and kidney). All infants' vital signs were monitored throughout and after the intervention and any significant event (apnea/bradycardia [respiratory pause of >20 seconds with consequent decrease in heart rate < 80], sustained bradycardia or tachycardia, sustained desaturation requiring intervention) was recorded.

### Statistical analysis

The sample size calculations were based on the following assumptions: i) a medium effect size of 0.50 (based on local agreement of clinicians as being clinically significant and based on previous literature); ii) statistical power of 80% and iii) a (2-sided) type I error of 5%. Considering a standard deviation of 4.7, a medium effect size would translate into a difference in PIPP score of 2.35 units. To account for technical or equipment problems, 10% was added to the sample size, thus 142 patients (71 per group) were required.

One planned interim analysis was conducted after half of the patients were enrolled. This analysis focused on the primary outcome and safety issues. Using the O'Brien-Fleming criteria for two-sided test, the alpha-level of this interim analysis was 0.005. The final analysis was conducted using a type one error of 0.048. The interim analysis did not reveal any statistically significant findings.

Descriptive statistics of baseline data for both groups were summarized. Pain scores were computed for every minute during the venipuncture. Difference in mean PIPP score during the first 4 minutes between treatment groups was assessed using independent Student's t-tests.

Differences between treatment and control groups in PIPP scores assessed over the first 4 minutes was compared using an analysis of variance (ANOVA) for repeated measures, adjusting for gender, postnatal age, use of sucrose and interaction between sucrose and treatment group. These variables may influence the expression of pain in infants [25,26].

Changes in physiological pain parameters such as heart rate, respiratory rate, and blood pressure were summarized using descriptive statistics. A log-rank test was used to assess whether or not the duration of cry was different for the group receiving tetracaine and the placebo group. Differences in number of attempts to obtain the blood work were determined using a Mann-Whitney test. For safety comparisons, descriptive statistics of local skin reaction, anomalies on the CBC, liver or renal function tests for each group were summarized and compared using Fisher's exact test.

## Results

**Figure 1:**
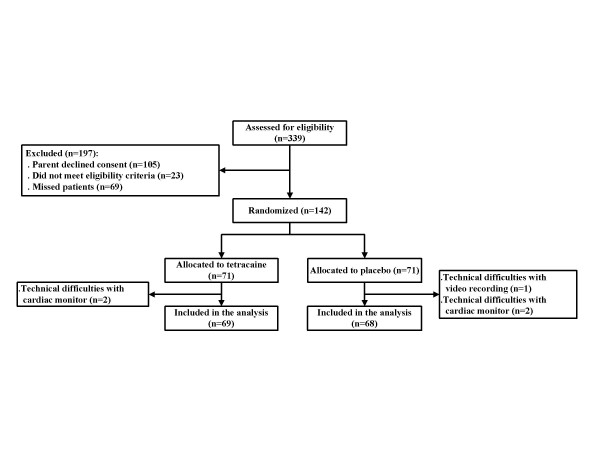
Participant flow through the study.

Infants were recruited for the study between January 2003 and December 2004. Three hundred and thirty nine infants were assessed for eligibility during that time. (Fig 1) 197 were excluded. Excluded infants were more mature and larger than included infants. (Table 1) This reflected the fact that more mature infants required minimal blood work. In total, 142 infants were randomized. Gestational age ranged from 24^0 ^to 41^4 ^week and birthweight from 556 to 4860 g. In five cases, problems with either the video recordings or the monitoring equipment precluded assignment of a primary outcome. Primary outcome was assessed in 137 infants (69 in the tetracaine and 68 in the placebo group).

Baseline characteristics of infants enrolled in the study were similar between groups (Table 2). The mean PIPP score in the first minute was 7.71 in the tetracaine group as compared to 7.62 in the placebo group (p = 0.909). No significant differences in PIPP scores during the 2^nd^, 3^rd ^and 4^th ^minutes were observed between the groups (Table 3). Median duration of cry in non-intubated infants was 5 seconds (tetracaine group) versus 0.5 seconds (placebo group) (p = 0.837). Additionally, 37 (57.8%) infants cried in the tetracaine group compared to 33 (50.0%) in the placebo group. Ease of venipuncture, number of attempts and success rates were similar between the groups (Table 3). Mean heart rate, respiratory rate, saturation and mean blood pressure at the end of baseline, 1, 2, 3, 4, 5, and 10 minutes were similar between groups (data not shown).

**Figure 2:**
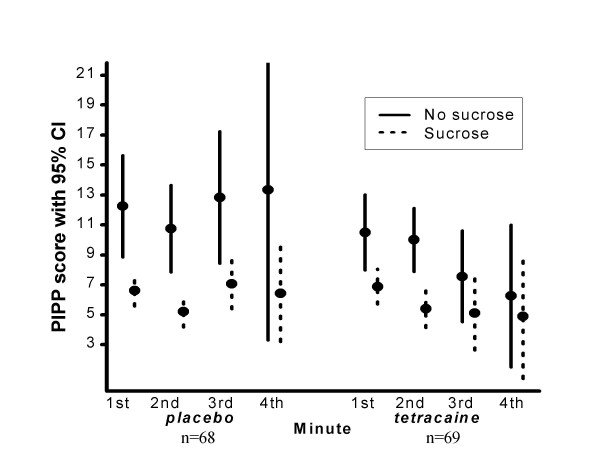
Post-hoc analysis, stratified by use of sucrose.

Previous RCTs suggest that local anaesthesia (EMLA) may not add further benefits to oral sucrose/glucose when used prior to a venipuncture [25,26]. We therefore conducted a post-hoc analysis of the primary outcome, stratified by use of sucrose (Fig 2). Fifty-four infants in the treatment group and 58 in the placebo group received sucrose. The repeated measures ANOVA, adjusted for gender, postnatal age and use of sucrose, indicated that use of sucrose was the only significant factor (p = 0.002) contributing to the variance in PIPP scores in the first 4 minutes of the intervention. PIPP scores were similar in infants who received sucrose, whether or not they also received tetracaine (interaction term p-value = 0.545).

Transient skin erythema was observed in 7 infants in the tetracaine group and in 4 infants in the placebo group (p = 0.532). No serious skin reaction was observed in either group. No significant changes were noted in any patients' CBC, ALT or creatinine in either group (data not shown). No significant adverse event was noted throughout the study. One infant in the placebo group died of fulminant necrotizing enterocolitis eight hours after randomization. After careful review of the event, the data safety monitoring committee and Health Canada concluded that this serious adverse event was unrelated to the study.

## Discussion

Topical tetracaine appeared safe but did not significantly decrease procedural pain in this group of preterm and term infants undergoing venipuncture. We had sufficient statistical power to observe a moderate effect size (difference of 2.35 on the PIPP score) between the treatment groups. Despite the statistical result, we believe our results are important clinically, for those looking after preterm and term infants requiring bloodwork.

To our knowledge, our study is one of the largest reported that includes preterm and term infants receiving tetracaine. Our results conflict with previous randomized trials using tetracaine for pain relief during venipuncture in newborns. For example, Jain et al, in a RCT that included 40 preterm and term infants reported a significant increase in the proportion of infants experiencing little or no pain (from 30% to 84%) during a venipuncture in the group receiving tetracaine, as compared to placebo [18]. A validated adaptation of the neonatal facial coding system and duration of cry were used to assess pain. This tool took into consideration only the facial expression of pain and the designation of little or no pain was made arbitrarily. Moore reported lower pain scores in the group that received tetracaine prior to venous cannulation in a RCT that included 40 preterm and term infants [19]. An unvalidated pain tool derived from several validated pain tools, which included facial expression, cry, heart rate and ease of cannulation was used. Our results are difficult to compare to other trials, as the pain tool and concomitant use of non-pharmacological pain interventions differed.

There are several explanations and limitations that could account for the statistically null results. Like other trials exploring ways to minimize pain in infants, this study was subject to potential problems related to the use of assessment processes and tools. Manually coding the videotapes and pain assessment is likely open to human error. Although measures were taken to decrease the chance of error, specifically, conducting random calibration exercises to verify the PIPP scores and testing interrater and intrarater reliability during the study, some margin of error remained. Likewise, the PIPP tool continues to undergo clinimetric testing and might result in refinement to its use and/or coding in the future.

The recommended dose and duration of application of tetracaine was used. Immature infants whose skin technically could absorb more medication rather than less would be expected to respond to the drug. Stability of the drug once removed from its original packaging has not been clearly established. We followed the manufacturer's recommendation at the time (and did not use the drug past 28 days after removal from its original package) (personal communication, Jason Collins, December 2002). As it becomes inactive, tetracaine crystallizes. We did not observe or feel crystals in the study gel during the trial.

Another limitation of this study is that we did not stratify according to the use of sucrose due to the timing of implementation of our oral sucrose guidelines. Sucrose has been shown to have a definite impact on the pain experienced during heel pricks and venipunctures in newborns 25 to 41 weeks gestation up to 28 days of age [26]. Given the proven benefits of sucrose, we felt it would have been unethical not to allow study infants to receive sucrose if they met the eligibility criteria, as infants not included in the study would have received it as standard of care.

A randomized trial comparing water, sucrose 24%, EMLA and EMLA with sucrose 24% in term newborns undergoing a venipuncture found that sucrose compared favourably with EMLA [27]. The concomitant use of EMLA did not increase further the analgesic efficacy of sucrose. In another RCT, glucose 30% was as effective as EMLA to decrease procedural pain in newborns undergoing venipuncture, as assessed by the PIPP [28]. Our own findings, along with those from these previous trials lead us to speculate that sucrose had an important impact on the pain scores in our study. These were confirmed by our post-hoc analysis, which showed that sucrose was the only significant factor included in the regression analysis contributing to the variance in PIPP scores in the first 4 minutes of the intervention. It is possible that sucrose was as effective as tetracaine at reducing procedural pain, but this remains a hypothesis, as our study design does not permit us to conclude this.

Several nurses, with varying degrees of experience, were involved in performing the venipunctures adding to the generalizability of our findings. However, given the sample size, this may have contributed to the lack of difference in the number of attempts and ease of procedure and possibly to the relatively low success rates of the venipuncture. Similarly, we included infants of all gestations, to assess effectiveness, rather than efficacy. The PIPP tool is validated for infants of all gestations and takes gestational age into account for scoring. However, higher or lower PIPP scores have been described in smaller infants [23,24]. Fifteen participants in our trial were less than 28 weeks gestation, thus the confounding effect was likely insignificant.

Finally, a venipuncture is more than a simple skin puncture. Infants are restrained, a tourniquet is applied and the procedure can last up to several minutes. It has been demonstrated in previous studies that handling and immobilization lead to behavioural and physiological reactivity [29,30]. Thus, the skin puncture alone is unlikely to be the only stressful (or painful) event during a venipuncture.

## Conclusion

Tetracaine did not significantly decrease procedural pain in infants undergoing a venipuncture, when used in a setting where sucrose is routinely used for procedural pain. Further studies, comparing sucrose, tetracaine and the two interventions combined are warranted, to better delineate which strategy is best to prevent pain associated with a venipuncture.

## Competing interests

The author(s) declare that they have no competing interests.

## Authors' contributions

BL designed the study, received peer reviewed funding to conduct the trial and drafted the manuscript. DH contributed to the study design, enrolled patients, collected data and had significant input into the drafting of the manuscript. IG participated in the design of the study, performed the statistical analysis and contributed to drafting the manuscript. RS participated in the design of the study and contributed to the drafting of the manuscript. CB participated in the design of the study. DM participated in the design of the study and in the drafting of the manuscript. All authors read and approved the final manuscript.

## Pre-publication history

The pre-publication history for this paper can be accessed here:



## Figures and Tables

**Table 1:** Characteristics of excluded and included patients

**Characteristics**	**Excluded (n = 174)**	**Included (n = 142)**	**Difference (95%CI)**	**P-value**
Birth weight in gms, mean (sd)	2475.98 (961.41)	2123.68 (951.74)	352.30 (139.34, 565.26)	0.001
Completed weeks of gestation, mean (sd)	35.10 (3.61)	33.21 (4.08)	1.89 (1.04, 2.74)	<0.001
Male, n (%)	90 (51.7)	85 (59.9)	-8.1 (-18.8, 2.9)	0.172

**Table 2:** Baseline characteristics

**Characteristics**	**Placebo (n = 71)**	**Tetracaine (n = 71)**
Birth weight in gms, mean (sd)	2116.14 (966.60)	2131.21 (943.47)
Completed weeks of gestation, mean (sd)	33.04 (4.28)	33.37 (3.89)
Male, n (%)	43(60.6)	42 (59.2)
Age in days, mean (sd)	6.58 (3.01)	6.77 (3.25)
Sucrose received (n)	58	54

**Table 3:** Study outcomes

**Outcome**	**n**	**Placebo**	**n**	**Tetracaine**	**Difference (95% CI)**	**p-value**
PIPP, 1^st ^minute, mean (sd)	68	7.62 (4.76)	69	7.71 (4.66)	-0.09 (-1.68, 1.50)	0.909
PIPP, 2^nd ^minute, mean (sd)	53	6.45 (4.32)	51	6.84 (4.42)	-0.39 (-2.09, 1.31)	0.650
PIPP, 3^rd ^minute, mean (sd)	25	8.44 (4.37)	26	5.96 (4.67)	2.48 (-0.07, 5.03)	0.056
PIPP, 4^th ^minute, mean (sd)	12	8.17 (5.08)	14	5.29 (5.10)	2.88 (-1.25, 7.01)	0.163
Duration of cry in seconds, Median (range)	66	0.5 (0, 175)	64	5 (0, 159)	0 (-3, 0)	0.837
Ease of insertion, median (range)	71	2 (1, 5)	71	1 (1, 5)	0 (0, 0)	0.456
Number of attempts	71	1 (1, 2)	71	1 (1, 2)	0 (0, 0)	0.414
Venipuncture successful, n (%)	71	36 (50.7)	71	46 (64.8)	-14.1 (-29.3, 2.1)	0.126
